# Correlation of retinal sensitivity in microperimetry with vascular density in optical coherence tomography angiography in primary open-angle glaucoma

**DOI:** 10.1371/journal.pone.0235571

**Published:** 2020-07-06

**Authors:** Katarzyna Zabel, Przemyslaw Zabel, Martyna Kaluzna, Aleksander Lamkowski, Damian Jaworski, Magdalena Wietlicka-Piszcz, Jakub J. Kaluzny

**Affiliations:** 1 Department of Sensory Organ Studies, Nicolaus Copernicus University, Collegium Medicum in Bydgoszcz, Bydgoszcz, Poland; 2 Oftalmika Eye Hospital, Bydgoszcz, Poland; 3 Department of Ophthalmology, Nicolaus Copernicus University, Collegium Medicum in Bydgoszcz, Bydgoszcz, Poland; 4 Department of Biostatistics and Biomedical Systems Theory, Nicolaus Copernicus University, Collegium Medicum in Bydgoszcz, Bydgoszcz, Poland; University of California San Diego School of Medicine, UNITED STATES

## Abstract

**Purpose:**

To evaluate the correlation between retinal sensitivity in microperimetry (MP) with vessel density (VD) using optical coherence tomography angiography (OCTA) in primary open-angle glaucoma (POAG).

**Methods:**

We enrolled 30 participants (52 eyes) with POAG and 15 participants (23 eyes) in the healthy control group. All participants were examined for retinal structure using OCTA to assess VD and Spectral domain OCT (SD-OCT) to assess ganglion cell complex (GCC) and peripapillary retinal nerve fiber layer (pRNFL) thickness. Retinal sensitivity was tested with MP and standard automatic perimetry (SAP).

**Results:**

The VD in moderate/severe POAG was lower than that in mild POAG and healthy control in the macular superficial vascular plexus (SVP) (38.7±6.3% vs. 42.9±5.2%, 49.7±2.6% respectively, P<0.001) and peripapillary radial peripapillary capillaries (pRPC) (36.4±5.7% vs. 43.6±6.6%, 49.1±2.4% respectively, P<0.001). The Pearson’s correlations between function-structure parameters were strongest with MP average sensitivity threshold and SVP VD in the area of whole macula (r = 0.68); followed by SAP mean deviation (MD) and pRNFL thickness (r = 0.63); SAP MD and pRPC VD (r = 0.59) and MP average threshold and GCC thickness (r = 0.54). We found the AUROCs for discriminating between glaucomatous and healthy eyes were highest for structural parameters as follows: pRNFL (0.94), macular SVP whole (0.92), pRPC (0.92) and GCC (0.91). Pairwise comparison of the above parameters showed no difference (P>0.05).

**Conclusion:**

The relationship between microvascular damage in the macular SVP whole and the decrease of MP average sensitivity threshold is stronger than the pRNFL thickness measurements and SAP parameters. OCTA and MP techniques are valuable methods that allow clinically monitor structural and functional changes in glaucomatous eyes.

## Introduction

Primary open angle-glaucoma (POAG) is a progressive optic neuropathy of the optic nerve characterized by retinal ganglion cell (RGC) apoptosis [1]. Increasing evidence indicates that abnormalities in retinal microcirculation and impairment of ocular blood flow have significant impact on development of POAG, but the exact role of vascular disorders in the pathogenesis of glaucoma has not been established [2–4].

Optical Coherence Tomography Angiography (OCTA) is an objective and non-invasive method of imaging the retinal and choroidal vessels. OCTA allows of repeatable, quantitative assessment of microcirculation in the macula and peripapillary [5, 6]. The reduction of retinal vascular density (VD) within the macula and optic nerve head (ONH) has been repeatedly confirmed in patients with POAG [7–11]. Takusagawa et al. observed with the use of projection-resolved OCTA focal capillary dropout in the macular superficial vascular plexus (SVP), but not the intermediate and deep vascular plexus (DVP) in glaucomatous eyes. Among the overall macular parameters, the SVP VD had the best diagnostic accuracy and was highly correlated with the corresponding retinal ganglion cell complex (GCC) thickness and visual filed (VF) sensitivity [12].

Standard automated perimetry (SAP) is most often performed to detect glaucomatous retinal function damage and is still the preferred method for assessing the 24° or 30° area of the VF center in patients with POAG. The macula, in which is most of the RGC, is located in the center of 5°-10° VF, which means that using the 24–2 SAP program, you might not notice the subtle changes in sensitivity in the macula that occur before defects appear in the peripheral parts of VF [13–16]. Microperimetry (MP) is a newer method of retinal function examination thanks to the use of eye-tracking system can measure retinal sensitivity at appropriate retinal points. During a single examination, it combines digital fundus photography and VF examination, along with an assessment of retinal sensitivity, allowing for the assessment of changes in the central part of the field of view, both in qualitative and quantitative terms [17].

The aim of our study was to determine whether the disturbances in retinal VD parameters are associated with the presence of retinal function abnormalities using MP and SAP. While there have been studies that looked at the relationship between function disorder and damage to the structure of the retina, to date, to our knowledge, the relationship between macular retinal sensitivity using MP and VD in OCTA has not been evaluated in patients with POAG. We hypothesized that the sites of reduced VD in the macula of the eyes with POAG have a lower retinal sensitivity measured by MP.

## Materials and methods

This was an cross-sectional study and included patients with POAG and a healthy control group. The protocol of the study was approved by the Bioethical Commission of Nicolaus Copernicus University in Torun, Collegium Medicum in Bydgoszcz. Approval number:600/2019. Each participant signed a voluntary consent form to participate. The examinations were conducted in accordance with the principles of the Helsinki Declaration between June 2019 and January 2020 at the Oftalmika Eye Hospital.

All patients included in the study had detailed ophthalmological examinations which included: best-corrected visual acuity (BCVA), slit-lamp biomicroscopy, dilated fundus examination, tonometry (Icare TAO1i, USA), pachymetry (Tomey EM-3000, Tomey Corporation, Japan), gonioscopy and axial length measurement (IOL Master 500, Zeiss Humphrey, Dublin, CA, USA). The thickness of the peripapillary retinal nerve fiber layer (pRNFL) and GCC were also measured using Spectral domain optical coherence tomography (SD-OCT), retinal VD assessment using OCTA, and VF and retinal sensitivity examination using SAP and MP were performed. These examinations were carried out over the course of one day, by the same ophthalmologist.

Patients with POAG were enrolled in the study based on the presence of features of glaucomatous optic neuropathy (diffuse or focal thinning of the neural retinal ring, ONH hemorrhagia, abnormal C/D ratio >0.6, C/D asymmetry between two eyes above 0.2), which was accompanied by a decrease in pRNFL thickness corresponding to the loss of VF in SAP with an open anterior chamber angle. Glaucoma was confirmed by the presence of repeated abnormal SAP results with a glaucoma hemifield test (GHT) outside normal limits or pattern standard deviation (PSD) outside the 95% normal limits or a cluster of 3 or more adjacent points in a typical localization for glaucoma. Patients with POAG were further classified into 2 groups based on the severity of VF damage in SAP; mild glaucoma was defined as mean deviation (MD) VF greater than -6dB, and moderate/severe glaucoma when MD was less than -6dB.

Healthy participants had intraocular pressures less than 21mmHg, normal ONH appearance without asymmetry, pRNFL thickness within normal limits, normal results in VF examination defined as a PSD within the 95% confidence limits and a GHT result within normal limits. The healthy control did not present with any ocular disease except for small refractive errors.

The general exclusion criteria were: age below 50 and above 85, BCVA≤0.6, refractive defect above ±3.0Dsph, IOP>23mmHg, ophthalmic surgery, with the exception of uncomplicated cataract phacoemulsification and uncomplicated anti-glaucoma surgery, when at least 3 months have passed since the surgery. People with vascular or non-vascular retinopathies, ocular or systemic diseases known to impair the VF, non-glaucomatous optic neuropathies and macular disease were also excluded.

### Optical coherence tomography angiography

The study used the Avanti Angiovue system (Optovue, Inc. Fremont, CA, USA), which provides non-invasive visualization of the retinal vascular network through the split-spectrum amplitude-decorrelation angiography (SSADA) algorithm. The Avanti RTVue XR device has the ability to scan at 70,000 A-scans/second and allows measurements with an axial resolution of 5μm using a light source with a wavelength of 840±10μm. The software version that was used, was the latest commercially available version (version 2017.1.0.151). The study was conducted on all of the patients between 12:00 and 16:00 after pupil dilation. The protocol that was used for macula analysis consisted of B-scans covering a 6x6mm area repeated horizontally and vertically. Each of the B-scans contained 400 A-scans with the center located at the fixation point. For peripapillary vessel analysis, a measurement that covered an area of 4.5x4.5mm centered on ONH, was made. The images also consisted of two sets of B-scans repeated horizontally and vertically, each consisting of 400 A-scans. The data were analyzed with commercially available software that enabled of automatic segmentation of SVP and DVP within the macula and the radial peripapillary capillaries (RPC) layer in ONH area. VD was calculated as the percent area occupied by flowing blood vessels in the selected region. In the macula, the VD analysis covered the entire test area of 6x6mm (VD whole), the parafoveal area between the rings 1mm and 3mm from the center of the fovea, and the perifoveal area between the rings 3mm and 6mm from the center of the fovea. The macular SVP was comprised between the inner limiting membrane (ILM) to outer boundary of the inner plexiform layer (IPL), while the DVP was comprised between outer boundary of the IPL to outer boundary of the outer plexiform layer. For the ONH scan, VD was analyzedon the entire surface of the en face image 4.5x4.5mm and in the peripapillary area—extending outwards from the ONH border measured in an elliptical area between 2 and 4mm. The RPC layer was defined as extending from the ILM to the posterior border of RNFL. For further analysis, an experienced ophthalmologist qualified good technical measurements with a scan quality(SQ) index of 6 or more on a 10-point scale with which a commercial device was equipped. The SQ index combines the previously used parameter the signal strength index (SSI), as well as an automated assessment of motion artifacts and defocus measure. Measurements with the presence of motion artifacts on en face images (irregular vascular patterns or blurred ONH border), and those with incorrect segmentation of individual plexuses were rejected.

OCTA is a modern imaging technique uses motion contrast for imaging of the vascular network enables qualitative and quantitative measurements of vessels at various retinal depths.

### Spectral domain optical coherence tomography

All patients underwent an SD-OCT examination to measure pRNFL thickness using a Spectralis OCT device (Heidelberg Engineering, Germany). The pRNFL was measured on the area of a circular scan that consisted of 768 A-scans. The scanning circle was 3.46mm in diameter and centered on the center of ONH. The global value of pRNFL thickness in the 360 degree range was analyzed. The GCC thickness assessment was carried out using an Avanti RTVue XR device camera. To measure the thickness of the retinal GCC, the built-in Avanti glaucoma module was used. The GCC scan was centered 1mm temporal from the fovea and covered a circular macula area of 6mm in diameter.

### Microperimetry

Each participant underwent an MP examination using the CenterVue MAIA device (CenterVue, Padova, Italy). The system is equipped with a scanning laser ophthalmoscope that provides accurate tracking and compensation of eye movement in real time. Tests were conducted prior to administering the mydriatics, in a quiet, dark room, and the lights were turned off after applying the veil to the untested eye. The target of fixation was a red ring with a diameter of 2° divided into four segments. The weak white background had a luminance level of 1.27cd/m2; the maximum stimulus intensity was 317.5cd/m2, which gives a dynamic range of 0-36dB; the stimulus size was Goldmann III; the stimulus duration was 200ms; and the test protocol was a 4–2 threshold strategy. The observer’s task was to press the button to indicate the presence of a light spot each time it was detected, while maintaining a central fixation. VF locations that required brighter stimuli to reach the threshold showed reduced sensitivity, and therefore lower dB values indicate lower retinal sensitivity, and conversely, higher dB values correspond to darkening stimuli and represent higher retinal sensitivity. The Expert Exam protocol was used in order to assess the average sensitivity threshold at various locations within the macula. The test grid consisted of 37 light points in a concentric area, 5° from the central fixation point, which means that a center area of 10° was tested, with 12 points on each concentric ring (2°, 6° and 10°) plus the measurement of one center point. In addition to assessing the sensitivity threshold of the retina, the device also gives the opportunity of estimating the index of macular integrity (a numerical value that describes the probability of a patient’s response to a stimulus to be normal, suspicious or abnormal compared to normative data corrected for age where higher number suggests greater likehood of abnormal findings) and records the stability of the fixation.

MP, also known as fundus related perimetry, includes a system to image the retina and an eye tracker to compensate eye movements during visual field testing. The device allows to precisely create a retinal sensitivity map of the quantity of light perceived in designated parts of the retina.

### Standard automated perimetry

SAP examination was performed using a Humphrey Field Analyzer II (Carl Zeiss Meditec, Dublin, CA, USA). Visual fields were acquired using the Swedish Interactive Threshold Algorithm (SITA) standard 24–2 strategies. Only reliable tests (≤33% fixation losses, ≤10% false-positives, and ≤10% false-negatives) VF without rim and eyelid artifacts; and cases with no evidence that the abnormal results were caused by diseases other than glaucoma were included. The severity of glaucoma was recorded as the MD value.

### Statistical analysis

The summary statistics for normally distributed continuous variables are presented as mean and standard deviation (SD) and as a median with interquartile range (IQR) for non-normally distributed variables. Categorical variables are presented as frequencies. Differences between continuous normally distributed variables were analyzed by the t test or by ANOVA together with the Bonferroni-type adjustment for multiple testing. In the case the data were not normally distributed, differences were tested by the Wilcoxon or Kruskal-Wallis test. When multiple patient groups were compared, multiple testing corrections were also applied. Differences for categorical variables were tested using the chi-square or Fisher exact test for independence. To compare the distributions of functional and structural parameters of eyes in various groups of patients, the Linear Mixed Effects model (LMM), which takes into account the correlation between repeated observations from the same individual, was used (with application of the inter-eye correlation). The models were also adjusted for age and SQ (where applicable). To study the association between the functional and structural parameters the LMM has also been used. The functional parameters were included in the models as the dependent variables, while the structural parameters, as well as the age of patients, were included as independent variables. The results were reported by the coefficient of determination (R^2^), which evaluates the proportion of variance in the dependent variable explained by the model. To assess the strength of the association between the structural and functional parameters, the Pearson’s correlation coefficient was calculated. Area under receiver operating characteristic curve (AUROC) was used to assess the diagnostic accuracy of considered variables to differentiate between healthy control and POAG eyes. The deLong’s or bootstrap test was used to compare the AUROC curves. The results were considered as statistically significant when the p-value was less than 0.05. The statistical analysis was performed with the use of the R-software, version 3.6.2 (packages lme4, gls, r2glmm and pROC).

## Results

Initially, 58 eyes of 30 participants with POAG, and 27 eyes of 15 participants from the healthy group who met the outlined inclusion and exclusion criteria, qualified for the study. However, of these, 52 eyes with POAG and 23 eyes from the healthy group were subjected to final analysis. 8 eyes were excluded due to poor image quality (motion artifacts, vitreous floaters, incorrect segmentation) in OCTA and SD-OCT examinations, and 2 eyes were excluded due to unreliable MP and SAP results. When both eyes of the same patient were included in the study, the correlation between the eyes was controlled in our analyses. Based on the degree of VF loss in SAP, 30 eyes of patients with POAG were assigned to the group with mild POAG (MD -2.6±1.6 dB) and 22 eyes were included in the group with moderate/severe POAG (MD -13.4±7.1dB). There was no significant difference in age, sex, hypertension, diabetes, central corneal thickness, or intraocular pressure between the POAG and healthy groups (Table 1). Among patients with POAG, at least one type of anti-glaucoma drops was used in 47 eyes (90.4%). In the mild POAG group, anti-glaucoma surgery was performed in 2 (6.7%) eyes, and in the group with moderate/severe POAG, in 3 (13.6%) eyes. We also assessed whether any of the drug classes was associated with any OCTA parameter by controlling the age and severity of the disease, however we did not find such impact.

**Table 1:** Demographic data and clinical characteristics of patients.

Parameter	Healthy	POAG		P Valueǂ
		Mild	Moderate/Severe	
**Number of eyes (patients)**	23(15)	30(20)	22(16)	
**Age (years)**	69±5.2	70.2±8.3	71.9±8.2	0.573
**Gender (Male/Female)**	6/9	8/12	9/6	0.429
**Self-Reported History of Hypertension (patients)**	8(53.3) †	9(45†	9(60) †	0.674
**Self-Reported History of Diabetes (patients)**	4(26.7) †	4(20) †	2(13.3) †	0.622
**Number of GlaucomaMedications**	0	1.5±0.9	2.1±1.1	0.049
**Eyes on Timolol**	0	13(43.3) †	13(59.1) †	0.401
**Eyes on Brymonidine**	0	3(10) †	8 (36.4) †	0.037
**Eyes on ProstaglandinAnalogs**	0	20(66.7) †	8(36.4) †	0.048
**Eyes on Carbonic Anhydrase Inhibitors**	0	10(33.3) †	16(72.7) †	0.011
**History of Glacuoma Surgery (eyes)**	0	2(6.7) †	3 (13.6) †	0.639
**BCVA (Snellen)**	1(1–1)*	1(1–1)*	1(1–1)*	0.270
**CCT (microns)**	540(532–549)*	537.5(497.5–564.5)*	517(486–540)*	0.485
**IOP (mmHg)**	17.8±2.1	18.3±2.1	17.6±2.5	0.552
**AxialLength (mm)**	22.9±0.7	23.2±1.0	23.37±0.8	0.296

Mean (standard deviation).

*Median (interquartilerange).

^†^n (%).

^ǂ^Statistical significance tested by ANOVA or Kruskal-Wallis test (for continuous variables) and by chi-square or Fisher exact test (for categorical variables).

Abbreviations: POAG = primary open-angle glaucoma; BCVA = best corrected visual acuity; CCT = central corneal thickness; IOP = intraocular pressure.

**Fig 1:**
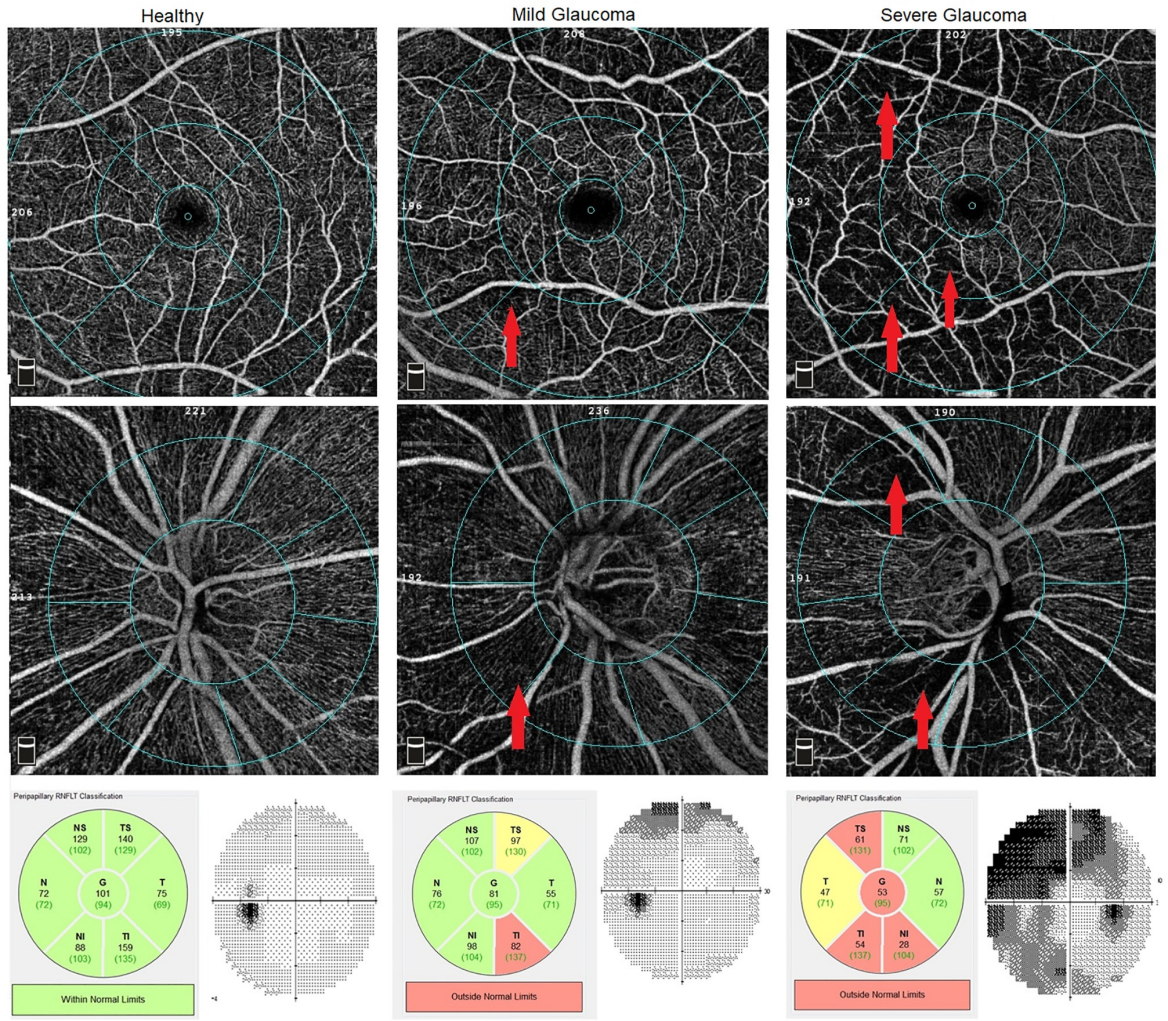
Optical coherence tomography angiography (OCTA) of vessel density (VD) map in healthy, mild and severe glaucoma. Top row and second row show the macular superficial l vascular plexus (SVP) (scan 6x6mm) and radial peripapillary capillaries (RPC), respectively. Bottom row: Peripapillary retinal nerve fiber layer (pRNFL) analysis from Spectral domain optical coherence tomography (SD-OCT) and standard automated perimetry (SAP) visual field (VF). The first column shows the healthy patient with normal VD. The second column: patient with mild glaucoma. Notable VD reduction at the inferonasal region of peripapillary RPC (pRPC) and perifoveal retina was observed (red arrows). pRNFL defects at the inferotemporal sector were noted on SD-OCT. pRNFL defects and capillary dropout area were compatible with superior VF scotomas observed in the SAP. Third column: In patient with severe glaucoma the superior arcuate scotoma and defects in inferotemporal quadrant are consistent with capillary dropout at the inferior and superior region of the pRPC and parafoveal and perifoveal retina (red arrows). The pRNFL evaluation shows abnormalities in the temporal and inferior quadrants.

**Fig 2:**
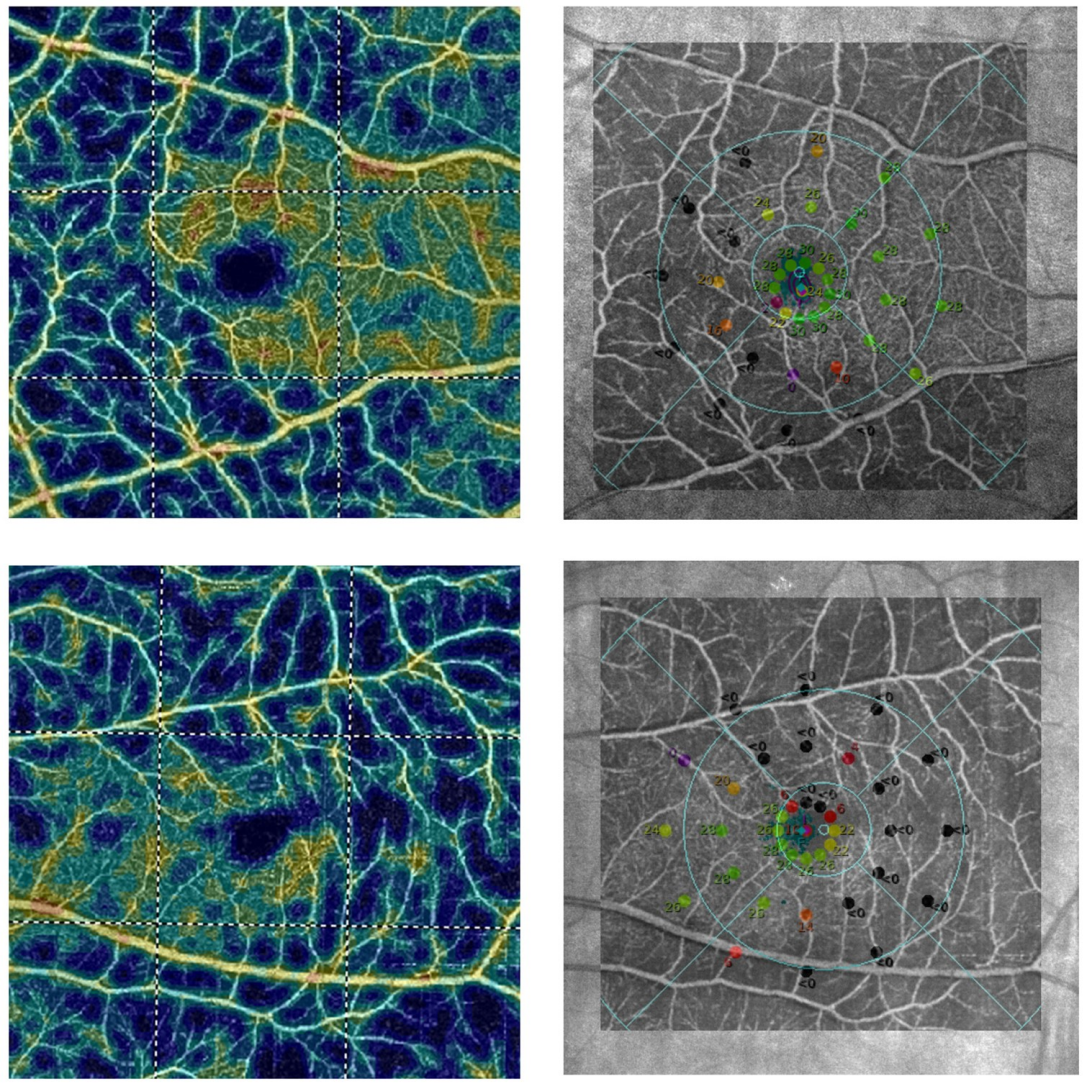
Relationship between sensitivity threshold points in the microperimetric (MP) examination of the retina and en face angiogram of superficial retinal vascular plexus (SVP) in the macula using optical coherence tomography angiography (OCTA). Top row: Right eye of female with primary open-angle glaucoma (POAG). Bottom row: Left eye of male with POAG. First column: The OCTA colour-coded vascular map of macular SVP. Cold colours on angiograms indicate capillary dropout areas. Second column: on the Scanning Laser Ophthalmoscope image with sensitivity threshold points in the MP examination, en face angiogram of macular SVP was superimposed to show the relationship between function and structure of the retina. Points at which sensitivity threshold was reduced or where there were deep scotoma points (black dots) in the MP, corresponded exactly to capillary dropout areas in OCTA.

Healthy eyes with normal pRNFL and GCC thickness had a clearly visible microvessel network in pRPC and SVP in the macula compared to eyes with POAG. As the disease progresses, the tendency to have a less frequent retinal microvessel network, and the appearance of sites where vascular flows are completely invisible, can be demonstrated (Fig 1). Comparing the results obtained in the angiograms of the macula with MP, it can be seen that the places where the density of the microvessels network in SVP was reduced, corresponded exactly to the areas where the retinal sensitivity threshold was decreased. Moreover, the capillary dropout places in the macular SVP corresponded to deep scotoma points (0dB) in the MP (Fig 2).

Quantitative analysis confirmed that the severity of POAG was significantly associated with deterioration of parameters of retinal function and structure. The evaluation of images obtained with OCTA showed significant reduction in retinal VD in pRPC and macular SVP whole in eyes with POAG. The moderate/severe POAG had significantly less VD in both the pRPC (36.4±5.7%) and macular SVP whole (38.7±6.3%) compared to the mild POAG (43.6±6.6%, 42.9±5.2%, respectively) and healthy group (49.1±2.4%, 49.7±2.6%, respectively) (P<0.001 for all pairwise comparisons, except VD comparison in macular SVP whole in eyes with mild POAG and moderate/severe POAG (P = 0.002)). Differences in VD in macular DVP between groups were not statistically significant. The DVP/SVP whole ratio in the healthy group was 1±0.1 and was significantly different from the mild POAG group (1.1±0.1), and the moderate/severe POAG group (1.2±0.1) (P<0.001). The SD-OCT study confirmed a significant reduction in pRNFL and GCC thickness in the eyes of POAG, and the fact that the degree of damage to the retinal structure increases with the severity of the disease (Table 2).

**Table 2:** Structural characteristics of the studied groups.

Parameter	Healthy	Mild POAG	Moderate/Severe POAG	P Values†		
				Mild vs Healthy	Moderate/ Severe vs Healthy	Moderate/ Severe vs Mild
**Optical CoherenceTomographyAngiography**						
**SVP whole VD (%)**	49.7±2.6)	42.9±5.2	38.7±6.3	0.001	<0.001	0.002
**SVP superior-hemi VD (%)**	50.2±3	43.3±5.3	39.1±6.1	0.001	<0.001	0.003
**SVP interior-hemi VD (%)**	49.6±2.7	42.5±5.2	38.4±6.6	<0.001	<0.001	0.003
**SVP parafovea VD (%)**	52.4±4.1	46.9±5.9	43.6±6.1	0.015	<0.001	0.018
**DVP whole VD (%)**	49.3±3.4	47.8±5.7	46.9±5.5	0.976	0.571	0.504
**DVP/SVP VD index**	1.±0.1	1.1±0.1	1.2±0.1	0.001	<0.001	0.011
**SQ Macula index**	8(7–8)*	7(7–8)*	7(7–8)*	0.208	0.162	0.782
**pRPC VD (%)**	51.2±2.9	43.6(6.6)	36.2±7.2	0.001	<0.001	<0.001
**RPC whole VD (%)**	49.1±2.4	42.7±5.6	36.4±5.7	0.001	<0.001	<0.001
**RPC superior-hemi VD (%)**	51.4±3.2	44.1±6.6	37.2±7.1	0.001	<0.001	0.001
**RPC inferior-hemi VD (%)**	51.1±2.9	43.1±6.9	35.1±8.1	0.001	<0.001	<0.001
**SQ OpticDisc index**	9(8–9)*	8(7–9)*	8(8–9)*	0.124	0.401	0.521
**Spectral Domain Optical Coherence Tomography**						
**GCC average (μm)**	98.2±9.8	82.3±10.9	71.6±11.3	<0.001	<0.001	0.001
**GCC superior (μm)**	97.4±1	82.7±11	71.6±10.8	<0.001	<0.001	0.001
**GCC inferior (μm)**	99.1±9.8	82.2±11.2	71.6±12.2	<0.001	<0.001	0.001
**pRNFLglobal (μm)**	101.8±10.3	78.8±13.9	61.4±14.2	<0.001	<0.001	<0.001
**pRNFL superior (μm)**	122.4±12.2	97.4±17.9	74.6±20.2	<0.001	<0.001	<0.001
**pRNFLinferior (μm)**	131±18.1	98.7±22.2	69.2±25.1	<0.001	<0.001	<0.001
**pRNFLtemporal (μm)**	72.4±17.6	57±15.9	49.4±11.9	0.003	<0.001	0.094
**pRNFLnasal (μm)**	81.6±11.7	62.1±15.2	52.2±15.6	<0.001	<0.001	0.027

Mean (standard deviation).

*Median (interquartilerange).

^†^P Values adjusted for age, inter-eye correlation and SQ (in OCTA), based on Linear Mixed Effects Model.

Abbreviations: POAG = primary open-angle glaucoma; SQ = scan quality; SVP = superficial vascular plexus; DVP = deep vascular plexus; p- = peripapillary; RPC = radial peripapillary capillaries; VD = vessel density; GCC = ganglion cell complex; RNFL = retinal nerve fiber layer; OCTA = optical coherence tomography angiography.

Assessment of retinal sensitivity in the MP showed that eyes with moderate/severe POAG had significantly worse parameters: average sensitivity threshold (20.2±5.4 dB) compared to eyes with mild POAG (25.3±2.3 dB) and the healthy group (26.7±1.4 dB) (P<0.001). Despite the fact that patients with mild POAG had lower average sensitivity threshold than healthy, the difference was not significant (P = 0.134). For the macular integrity index, a significant difference was only between moderate/severe POAG (92.2±15.4) and healthy (71.6±20.2) (P = 0.005). In the 24–2 SAP, VF testing in eyes with moderate/severe POAG showed significant differences in VFI (62.1±27%) and MD (-13.4±7.3dB), and the group with mild POAG (95.8±3.6%, -2.6±1.5dB) and healthy (98±1.5%, -1.8±1.5 dB, respectively) (P<0.001). There were no significant differences in retinal function parameters between eyes with mild POAG and healthy control (Table 3).

**Table 3:** Functional characteristics of the studied groups.

Parameter	Healthy	Mild POAG	Moderate/Severe POAG	P Values†		
				Mild vs Healthy	Moderate/ Severe vs Healthy	Moderate/ Severe vs Mild
**Average sensitivity thershold (dB)**	26.7±1.4	25.3±2.3	20.2±5.4	0.134	<0.001	<0.001
**Macular integrity index**	71.6±20.2	80.2±21.5	92.2±15.4	0.173	0.005	0.07
**VFI (%)**	98±1.5	95.8±3.6	62.1±27	0.573	<0.001	<0.001
**MD (dB)**	-1.8±1.5	-2.6±1.5	-13.4±7.3	0.431	<0.001	<0.001
**PSD (dB)**	2.4±0.6	2.8±1.6	8.6±3.7	0.221	0.023	0.187

^†^P Values adjusted for age and inter-eye correlation based on Linear Mixed Effects Model.

Abbreviations: POAG = primary open-angle glaucoma; VFI = visual field index; MD = mean deviation; PSD = pattern standard deviation.

**Fig 3:**
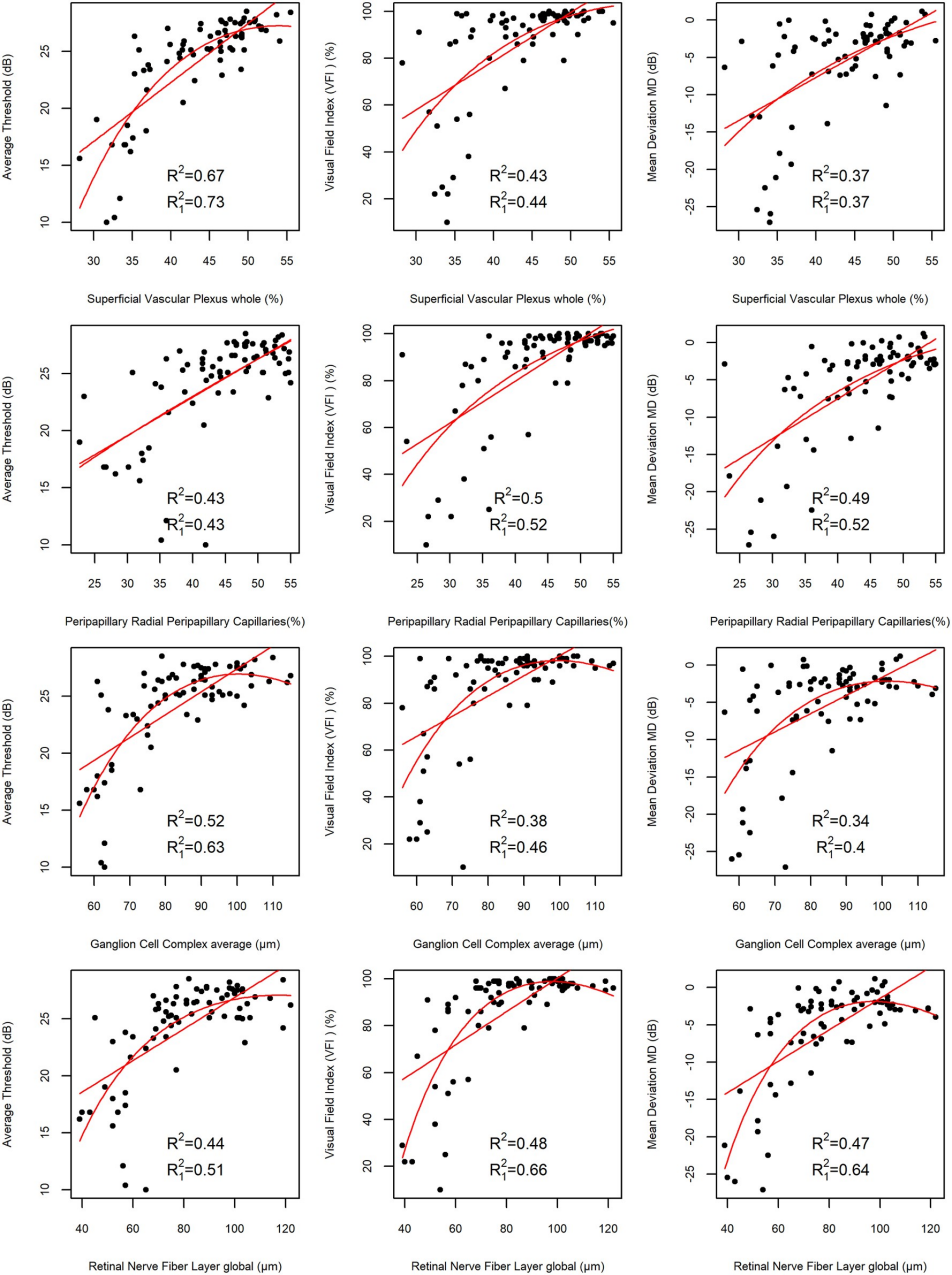
Scatter plots illustrating the correlation between structural and functional parameters with linear and quadratic regression curves. The first column shows dependence between average sensitivity threshold and macular superficial retinal vascular plexus whole(SVP whole), average sensitivity threshold and peripapillary radial peripapillary capillaries (pRPC), average sensitivity threshold and ganglion cell complex (GCC), average sensitivity threshold and peripapillary retinal nerve fiber layer (pRNFL). The second column illustrates correlation between visual field index (VFI) and macular SVP whole, VFI and pRPC, VFI and GCC, VFI and pRNFL. The third column shows dependence between mean deviation (MD) and macular SVP whole, MD and pRPC, MD and GCC, MD and pRNFL. Additionally the values of the adjusted coefficient of determination for the linear (R^2^) and quadratic dependence (R_1_^2^) for the depicted variables, obtained from the LMM fit adjusted for age, inter-eye correlation and SQ (where applicable) are shown.

In order to examine the correlation between retinal function assessed in MP and SAP, and retinal structure analyzed using OCTA and SD-OCT, the values of partial correlations corrected for age, inter-eye correlation and SQ (in OCTA) were determined. The analysis showed that the strongest correlation occurs between SVP whole VD in the macula and average sensitivity threshold (Pearson’s r = 0.68, P<0.001). Strong correlations also existed between pRNFL thickness and MD, pRNFL thickness and VFI (Pearson’s r = 0.63, r = 0.61, P<0.001, respectively), while there were moderately strong correlations between pRPC VD and MD (Pearson’s r = 0.59, P<0.001), pRPC VD and VFI (Pearson’s r = 0.58, P<0.001), pRPC VD and PSD, GCC thickness and average sensitivity threshold (Pearson’s r = 0.59, r = -0.55 r = 0.54, respectively, P<0.001) (Table 4). The dependence between the functional and structural parameters has also been studied with LMM (Fig 3). The highest values of the coefficient of determination have been obtained for the model displaying the dependence between SVP whole VD in the macula and MP average sensitivity threshold:R^2^ = 0.67 for the linear- and R_1_^2^ = 0.73 for the curvilinear dependence, which indicates that about 67% and 73% respectively, of variation in average sensitivity threshold is explained by the model.

**Table 4:** Pearson correlation coefficient between structure and function of the retina.

Parameter	Average sensitivity threshold	Macular integrity index	VFI	MD	PSD
**SVP whole VD**	0.68 (<0.001)	-0.33 (0.004)	0.47 (<0.001)	0.42 (<0.001)	-0.44 (<0.001)
**DVP whole VD**	0.34 (0.003)	-0.16 (0.166)	0.2 (0.082)	0.166 (0.156)	-0.03 (0.766)
**DVP/SVP VD index**	-0.44 (<0.001)	0.27 (0.021)	-0.33 (0.004)	-0.31 (0.007)	0.45 (<0.001)
**pRPC VD**	0.48 (<0.001)	-0.31 (0.008)	0.58 (<0.001)	0.59 (<0.001)	-0.55 (<0.001)
**GCC average**	0.54 (<0.001)	-0.25 (0.029)	0.47 (<0.001)	0.47 (<0.001)	-0.3 (0.009)
**pRNFLglobal**	0.47 (<0.001)	-0.26 (0.024)	0.61 (<0.001)	0.63 (<0.001)	-0.38 (0.001)

Pearson’s r (P Value) adjusted for age, inter-eye correlation and scan quality (in OCTA).

Abbreviations: VFI = visual field index; MD = mean deviation; PSD = pattern standard deviation; SVP = superficial vascular plexus; DVP = deep vascular plexus; pRPC = peripapillary radial peripapillary capillaries; VD = vessel density; GCC = ganglion cell complex; pRNFL = peripapillary retinal nerve fiber layer; OCTA = optical coherence tomography angiography.

The AUROC was used to reflect the diagnostic accuracy of structural parameters of the retina obtained using OCTA and SD-OCT imaging (Table 5). To distinguish healthy eyes from eyes with POAG, the highest diagnostic accuracy was obtained for pRNFL, macular SVP whole, pRPC, GCC (AUROC 0.94, 0.92, 0.92, 0.91, respectively). The pRPC VD, pRNFL thickness and macular SVP whole VD showed the highest sensitivity at 95% specificity (0.79, 0.77, 0.74, respectively). Pairwise comparisons showed that diagnostic accuracy among all structural parameters did not differ significantly.

**Table 5:** Area under the receiver operating characteristic curve and diagnostic sensitivity of the structural to diagnose primary open-angle glaucoma.

Parameter	AUROC (95% CI)	Sensitivity (95%specificity)	P Value for pairwise AUROC comparison*			
			SVP whole VD	DVP/ SVP index	pRPC VD	GCC average
**SVP whole VD**	0.92 (0.85–0.98)	0.74	-	-	-	-
**DVP/SVP VDindex**	0.89 (0.81–0.96)	0.65	0.526	-	-	-
**pRPC VD**	0.92 (0.86–0.98)	0.79	0.827	0.353	-	-
**GCC average**	0.91 (0.83–0.98)	0.5	0.8	0.658	0.645	-
**pRNFLglobal**	0.94 (0.9–0.99)	0.77	0.4	0.079	0.299	0.147

Sensitivities at 95% specificity were evaluated.

* DeLong’s or bootstrap test.

Abbreviations: AUROC = area under the receiver operating characteristic curve; CI = confidence interval; VFI = visual field index; MD = mean deviation; PSD = pattern standard deviation; SVP = superficial vascular plexus; DVP = deep vascular plexus; pRPC = peripapillary radial peripapillary capillaries; VD = vessel density; GCC = ganglion cell complex; pRNFL = peripapillary retinal nerve fiber layer.

## Discussion

The results of this study confirmed our hypothesis showing a significant relationship between the assessment of retinal sensitivity using MP and the loss of retinal microvascular network in the OCTA examination. The strongest correlation was between average sensitivity threshold decrease and loss of microvascular density in macular SVP whole.

Diagnosis and monitoring of glaucoma in clinical practice is based on the assessment of functional and structural parameters of the retina, and pRNFL thickness analysis using SD-OCT and VF assessment using SAP has been the standard so far [18]. However, there are some limitations to these methods, which results in the search for new diagnostic markers. Jia et al. were the first to prove that OCTA could be useful in glaucoma diagnosis. They found that eyes with an early form of glaucoma in relation to a healthy group have a lower ONH VD and a reduced disc flow index [19, 20]. Since then, many scientific reports have confirmed the usefulness of OCTA imaging in the diagnosis of glaucoma, and have shown that the reduction of VD in patients with glaucoma also occurs in the peripapillary area and macula [21–24]. Liu et al. found that the peripapillary flow index and peripapillary VD are significantly lower in glaucomatous eyes and showed a correlation with SAP. Moreover, the disc flow index proved to be an unreliable parameter because of the absence of an OCT signal in segments of major blood vessels [25].

The development of OCTA software has enabled the quantification of angiograms, along with more accurate segmentation. The microcirculation assessment in RNFL has become possible, which reflects the RPC plexus formed by a unique network of capillary microvessels that are playing a key role in meeting the nutritional requirements of RGC axons [26]. Yarmohammadi et al. used OCTA to assess VD in the RPC layer in glaucomatous eyes. They were the first to demonstrate that circumpapillary VD and whole en face image VD in the RPC layer were significantly lower in eyes with POAG compared to healthy and glaucoma suspected [24]. Subsequent reports confirmed that patients with POAG have a decrease of VD in RPC, which may correlate with the loss of retinal function in SAP [27–32].

We showed that the correlation of structural parameters in the peripapillary area (pRNFL thickness and pRPC VD had a stronger relationship with SAP parameters than with MP parameters. This is probably due to the function of RGC axons and the associated microvessel network, which in the peripapillary area, come from different regions of the retina where the cell bodies of RGCs are located. Therefore the observed changes of pRNFL thickness reduction may result in damage to retinal function in the remote area. In view of this, it seems that a larger area used to test retinal function in 24–2 SAP corresponds better with structural peripapillary indicators in glaucoma than MP does. The functional parameters that correlated best with pRNFL thickness and pRPC VD, in our study, were MD and VFI, while MP average sensitivity threshold showed weaker correlation. Yarmohammadi et al. were the first to evaluate the correlation between the severity of retinal function damage in the 24–2 SAP examination and the loss of RPC VD using OCTA in POAG patients. They showed that linear associations between SAP MD with RPC VD was significantly stronger than the relationship between SAP MD and RNFL thickness [33]. The results of our research also showed linear associations between loss of pRPC VD and SAP MD damage, which did not differ significantly from the correlation between SAP MD and pRNFL thickness. The reason for the difference in the obtained results may be the use of a different device to assess the thickness of the pRNFL.

Contrary to peripapillary parameters, structural indicators of the retina in the macula (SVP whole VD, GCC thickness) had a higher correlation with the study of retinal function tested by MP. This is because MP focuses only on the central macular area where most of the RGC is located, and mostly coincides with the analyzed area in the SVP whole. We showed that VD in macular SVP whole compared to GCC thickness has a higher correlation with MP average sensitivity threshold, which may indicate the special role of vascular mechanisms in the pathogenesis of glaucoma. Our research also showed that peripapillary parameters show a stronger correlation with SAP than macular parameters, as confirmed by other authors [34]. This may be because the macula, in which most of the RGC is located, is represented in the center of 5°-10° VF, whereas the standard 24–2 SAP program is tested only by 8–16 points (out of 54). Test points spaced 6° apart in 24–2 SAP may be an inaccurate test when assessing macular sensitivity [13–15]. In SAP, in addition to programs 30–2 and 24–2, it is also possible to examine a 10° VF center with a higher resolution, i.e. with 64 test points spaced 2° apart. It is suggested that using 24–2 SAP, you might not see the changes in the macula before defects appear in the peripheral parts of VF. Therefore, for this purpose, the VF test can be used, which assesses the central 10° VF better than the 24–2 SAP program [16, 35]. Penteado et al. found that the relationship between 10–2 SAP mean sensitivity and GCC thickness was stronger than with macular SVP VD [36]. In contrast, our results showed a higher correlation between SVP whole VD and MP average sensitivity threshold, which could be due to the difference in the size of angiograms. The small scanning area they used (3x3mm, which is unlike our study where the scanning area was 6x6mm) could result in the overlooking of significant microvascular pathology in glaucoma, located peripherally outside the scan area. Additionally, the obtained angiograms were smaller than the area covered by the 10–2 SAP program, which might have influenced the strength of the structure-function relationship that was found.

A new method of assessing visual function fundus-driven perimeters has emerged in recent years, known as microperimetry. Previously, Scheifer et al. used fundus-oriented perimeters using individually condensed test grids and found increased detection of VF glaucoma lesions in morphologically visible areas of the retina in the macula and when test points are located close to each other, compared to conventional automated perimetry [37]. Studies have confirmed the usefulness of MP in assessing retinal function in eyes with glaucoma, and showed a significant relationship between the reduction in retinal sensitivity in the MP examination and the decrease in GCIPL thickness [38–43]. Lima et al. showed that retinal sensitivity in MP examination not only significantly correlated with retinal sensitivity in the 10–2 SAP study, but also detected retinal areas with reduced sensitivity in quadrants where normal results were obtained in 10–2 SAP. The reduced retinal sensitivity in these areas corresponded to a reduction of up to 75% in spot thickness in the OCT imaging [44]. We found no reports assessing the relationship between retinal sensitivity using MP and vessel density in OCT-A examination.

Based on the AUROC interpretation, we have shown that structural parameters obtained using OCTA (VD in macular SVP whole and in pRPC and using SD-OCT (thickness of pRNFL and GCC) have high diagnostic ability (AUROC>0.9 for all parameters), and pairwise comparison revealed that they have the same diagnostic power performance, which was confirmed in numerous studies [25, 27–34]. In the current study patients with mild glaucoma have lower VF parameters than healthy, however we believe that due to the early stage of the disease these changes were not statistically significant. Defects in VF appear only when about 30% of RGC is damaged, and retinal structural changes precede the occurrence of functional abnormalities in patients with glaucoma [45, 46]. In studies where the diagnostic capabilities of functional parameters were compared using MP with structural parameters, the advantage of structural assessment in glaucoma diagnostics was also demonstrated [47–49]. Contrary to our results and other reports, a study by Rao et al. showed that the diagnostic ability to distinguish healthy eyes from glaucoma eyes was much stronger for structural parameters obtained using SD-OCT such as pRNFL and GCC thickness than for VD measurements of the ONH, pRPC and SVP on OCTA [50]. The reason for which they obtained poor results of diagnostic accuracy of VD parameters in ONH and pRPC may be because of lower SSI of the analyzed angiograms. Images with a higher SSI value give better clarity and segmentation, which results in better repeatability, and additionally this may affect the quantitative indicators [51]. In the macular density analysis, low AUROC value for SVP could be due to the 3x3mm scanning area, which may result in overlooking significant RGC pathology and peripheral microvessels outside the scanning area. Similar results of AUROC analysis were obtained by Penteado et al. who also used the OCTA scan size 3x3mm. Diagnostic accuracy of structural parameters in SD-OCT and OCTA was higher for GCC thickness than VD to distinguish between glaucoma and healthy eyes. They obtained the opposite results when distinguishing between glaucoma suspect eyes and healthy eyes. The diagnostic accuracy of the VD was better than the GCC thickness suggesting that detectable changes in the form of a reduction in VD within macula may precede the loss of RGC in glaucoma [36].

We found no significant difference in VD in macular DVP whole between the healthy and POAG groups as confirmed by other reports [12]. Glaucoma primarily affects RNFL, GCL and partly IPL layers that are supplied by SVP, while DVP supplies blood to the middle layers of the retina that do not contain RGCs [52, 53]. When analyzing DVP, there are concerns of projection artifacts caused by superficial vessels casting shadows on the deeper layers of the retina, which may affect the obtained results. Despite the fact that we are using the latest version of the software equipped with the AngioVue three-dimensional Projection Artifact Removal (3D PAR) algorithm, projection artifacts in the deeper layers of the retina are still noticeable, which can affect the results obtained in each group. Due to this and the lack of normative values for OCTA results, we calculated the DVP/SVP whole ratio. We have shown that as the glaucoma progresses, the DVP/SVP whole ratio increases, as in HC it was 1, in mild POAG it was 1.1, and in moderate/severe POAG it was 1.2. The results of this analysis are consistent with our previous study where the DVP/SVP whole ratio in patients with Alzheimer’s disease (AD) was the lowest and differed significantly from the healthy and POAG groups [54]. In our opinion, this can be a useful parameter in the event of an unexplained reason for decreased pRNFL thickness. The results suggest a different vascular phenotype in POAG than in AD where microvascular damage was noticeable in macular DVP and differed from that in POAG.

Several limitations of the current study should be considered. The first limitation was the relatively small number of subjects. The second was the inclusion of both eyes of subjects for the analysis. However, we accounted for the correlation between the two eyes of subjects using validated statistical methods. The severity of glaucoma was determined on the basis of the severity of VF damage in SAP, which could have an impact on the diagnostic accuracy of the other parameters. In addition to glaucoma, VF parameters are also affected by refractive errors or lens opacities. Another limitation was the lack of a normative database for MP to recognize and determine the severity of glaucoma. Due to the lack of a normative database, we were not able to classify patients with glaucoma and assess clinically significant dark spots in MP and compare the results with SAP. Additionally, the possible effects of various systemic diseases or drugs, blood pressure and perfusion pressure on retinal VD have not been determined. Patients in the POAG group used anti-glaucoma drugs that were not discontinued, but we found that none of the drug classes was related to the OCTA parameter that was analyzed. The cross-sectional nature of the study limits the determination of the temporal relationship between loss of vessel density in OCTA and glaucomatous structural and functional damage to the retina.

In summary, the quantitative and qualitative angiograms analysis of the SVP in the macula and pRPC layer showed decline VD in patients with more severe POAG, and the reduction in MP average sensitivity threshold corresponds to the capillary dropout areas in SVP. The links between microvessel damage in macular SVP and average sensitivity threshold decrease in MP are stronger than standard analyses, such as pRNFL thickness and VF assessment in SAP. Comparing the correlation results, in the case of structural assessment of the macula (GCC thickness, SVP whole VD), stronger relationships occur with MP average sensitivity threshold, while peripapillary parameters (pRNFL thickness, pRPC VD) correlate stronger with SAP. This is because testing retinal function in the macula using MP involves testing a larger number of points in the central 10° area, as well as real-time eye movement compensation, which means that the test points are located at pre-determined retinal locations. For these reasons, OCTA and MP are promising technologies that enable us to clinically monitor structural and functional changes in glaucoma. These methods can also potentially contribute to a better understanding of the pathophysiology of this disease, but require further research in this direction.

## Supporting information

S1 Dataset (XLSX)
